# Endovascular approaches in pediatric interventional oncology

**DOI:** 10.1186/s42155-020-00190-7

**Published:** 2021-01-02

**Authors:** Raja Shaikh, Fernando Gomez Munoz

**Affiliations:** 1 Department of Radiology, Boston Children’s Hospital and Harvard Medical School, 300 Longwood, Boston, MA-02115 USA; 2 Hospital Clinic-Hospital Sant Joan de Deu, C/ Villarroel 170, Passeig de Sant Joan de Déu, 2, Esplugues del Llobregat, 08950 Barcelona, Spain

**Keywords:** Interventional oncology, Pediatric tumors, Children’s oncology, Hepatocellular carcinoma, Hepatoblastoma, Neuroblastoma, Sarcoma, Retinoblastoma, Tumor bleeding, Tumor embolization

## Abstract

The demand for interventional oncological (IO) treatment of pediatric cancers is becoming increasingly common, at least at several tertiary care institutions. The data and techniques used in pediatric IO are largely extrapolated from experience in adult patients. The management of pediatric tumors differs from that in adults in several categories, such as, the curative intent of treatment, wide use of general anesthesia, aggressive pain management, potentially longer hospital stay, variation in chemotherapy dosing etc. Additionally, pediatric cancers are managed by protocols directed by national and international oncology groups such as the Children’s Oncology Group (COG). Consequently, the translation and adoption of these techniques is gradual, but there is a noticeable uptrend due to the growing need. This review will update the current endovascular IO treatments for common pediatric liver, renal, bone and soft tissue tumors.

## Background

Pediatric interventional oncology (IO) practice is mainly based off the adult IO experience, although there exist inherent differences between the two groups. While cancers occurring in adults are classified by the anatomical site of the primary tumor, cancers in children and younger adolescents are classified by histology (tissue type) into 12 major groups using the International Classification of Childhood Cancers (ICCC). Extracranial malignant pediatric solid tumors represent 52% of cancers in the 15- to 19-year-old-age group with germ cell tumors being the most common. In younger children, embryonal cancers, retinoblastoma, neuroblastoma, and hepatoblastoma, are more prevalent. The most common malignant solid tumors in adolescents are extracranial germ cell tumors (GCTs), bone and soft tissue sarcomas, melanoma and thyroid cancer (Allen-Rhoades et al. 2018).

Catheter directed chemotherapy regimens are relatively new and unfamiliar in pediatric setting. The decision making process often demands close discussion involving all care givers. Due to the developing physiological processes, adult chemotherapy dosing, cannot be applied in children as this may cause variable medication exposure, clearance, toxicity and unpredictable efficacy. Body surface area (BSA) based chemotherapy dosing used in adults can greatly overestimate the dose in younger children, where body weight may more accurately predict drug exposure (Leahey 2012; Veal and Boddy 2012). These variations warrant careful considerations and treatment planning. Use of general anesthesia, support from child psychology in preparation for invasive procedures is common in pediatric practice unlike that in adults (Temple and Marshalleck 2014). Arterial spasm and dissection are more common in children which may impede very distal selective catheterization and use of large caliber catheters. Post-embolization syndrome is also more common in children and often needs good supportive care and longer hospital admission (Temple and Marshalleck 2014; Bissler et al. 2002). Despite these impediments, there is an evident increase in the interest for these techniques. An example of this is a comparison between the number of indexed publications on interventional oncology for the management of pediatric malignancies in PubMed. Between 2010 and 2014, there were less than 180, and between 2015 and 2019, there were greater than 450 relevant publications. Percutaneous biopsy, percutaneous ablation, bland embolization, chemoembolization, radioembolization, combined percutaneous therapies or combination of systemic plus locoregional therapies are becoming more common among pediatric oncological treatments and represents an opportunity for interventional radiologists. This MEDLINE based review used relevant keywords to identify current literature in endovascular treatment of cancers in children.

## Liver tumors

Hepatoblastoma and hepatocellular carcinomas (HCC) are the predominant primary liver malignancies (1% of pediatric malignancies) in children. Hepatoblastoma accounts for two thirds of malignant liver tumors in children. Other liver malignancies in children include hepatocellular carcinoma, sarcomas, germ cell tumors and rhabdoid tumors. Benign tumors of the liver in children include vascular tumors, hamartomas and adenomas (Litten and Tomlinson 2008). In contrast to adults, HCC in children occur in normal livers without underlying cirrhosis, and a smaller proportion occur in the background of chronic liver diseases (Lungren et al. 2018). Staging and treatment decisions for pediatric liver tumors are based on guidelines from the three principal international cooperative groups that study childhood liver cancer: Children’s Oncology Group (COG), Société Internationale d’Oncologie Pédiatrique Epithelial Liver group (SIOPEL), and the Japanese Pediatric Liver Tumor Study Group (JPLT). The PRETEXT (PRETreatment EXTent of disease) and POST-TEXT, proposed by SIOPEL, is used in staging of pediatric HCCs.

### Bland embolization

In liver, bland embolization has been used to treat hepatic adenomas (Deodhar et al. 2011), ruptured hepatocellular carcinomas (Yamada et al. 2015), hepatoblastomas (in preparation for surgery) (Krauel et al. 2009), focal nodular hyperplasia (Oliveira et al. 2015) and vascular tumors such as hemangiomas (Sun et al. 2015; Kullendorff et al. 2002). Due to smaller blood volume in children, especially infants, uncontrolled blood loss can prove catastrophic during resection or tumor rupture, especially with hypervascular tumors. A variety of embolic agents can be used such as poly vinyl alcohol (PVA) microparticles of varying sizes, tris-acryl gelatin microspheres (Embosphere, Biosphere Medical, Rockland, MA), pledgets of gelatin sponge (Gelfoam, Pfizer, New York, NY, USA) and microfibrillar collagen (Tsochatzis 2014; Kishore 2017). Typically, particle sizes 100–300 μm are favored because smaller particles can cause biliary necrosis and bilomas and larger particles do not cause sufficient tumor ischemia (Lungren et al. 2018). Liquid embolization agents include *n*-butylcyanoacrylate (nBCA; Trufill nBCA Liquid Embolic, Codman Neurovascular Inc., Raynham, MA, USA), ethyl vinyl alcohol polymer (Onyx, eV3 Endovascular Inc., Irvine, CA, USA) and ethanol. Occlusion of large arterial feeders may require use of pushable or detachable coils. Often a combination of these agents may be used.

### Transcatheter arterial chemoembolization (TACE)

**Fig. 1:**
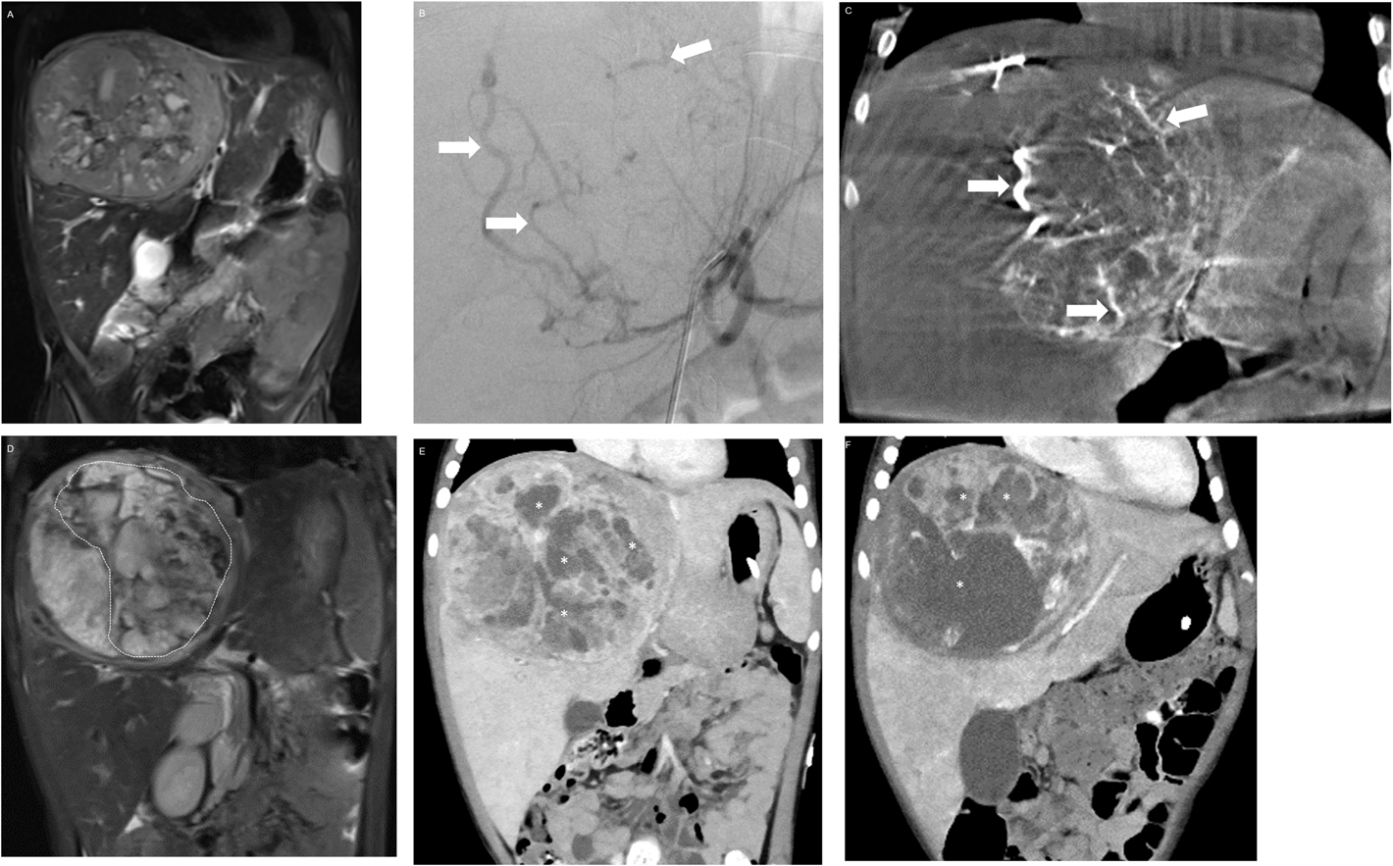
T2- weighted fat saturated coronal (**a**) MR image demonstrating a large hepatocellular carcinoma in a 2-year-old male patient which was unresponsive to systemic chemotherapy. **b** Angiography obtained during transcatheter arterial chemoembolization demonstrating several hepatic arterial branches encasing and supplying the tumor (arrows). These feeders were injected with 30 mg of doxorubicin emulsified with lipiodol followed by embolization with 150–250-μm poly vinyl alcohol particles confirmed on cone-beam computed tomography (**c**) obtained post embolization demonstrating the radio-opaque embolization beads in the tumor bed (arrows). **d** Post embolization T1- weighted post contrast coronal MRI obtained 3 weeks later demonstrating heterogeneous necrotic area (dotted line) within the tumor. Coronal computed tomography obtained 3 weeks (**e**) after the first TACE procedure and 3 weeks (**f**) after the second TACE procedure demonstrating increasing necrotic areas within the tumor (asterisk). The patient underwent a successful liver transplant 6 weeks later

This procedure increases the intratumoral concentration of the chemotherapeutic medication (most commonly doxorubicin) with the embolization aspect increasing the dwell time of the medication inside the target tissue while reducing the amount of drug entering the systemic circulation. The hypoxia and ischemia induced by TACE further augment the chemotherapy dose achieved within the neoplastic tissue. Chemoembolization has been utilized as neoadjuvant or to treat non-resectable liver hepatoblastoma (Hishiki 2013; Vogl et al. 2006; Tan et al. 2014) or hepatocellular carcinoma (Arcement et al. 2000). Recently, in a series of 8 patients (4–17 years) with unresectable HCCs, there was change in tumor volume by 51%, change in alfa fetoprotein level by − 49.6%, successful bridge to orthotopic liver transplantation with mean interval of 141 days (Weiss et al. 2018). TACE has also been advocated as a bridge therapy for liver transplantation (Hishiki 2013; Weiss et al. 2018; Li et al. 2008) (Fig. 1). In simple setting, TACE can be performed as an emulsion of the chemotherapeutic with an oil-based contrast medium such as ethiodized oil. Increasingly, vendor supplied, size specific drug-eluting beads (DEB 100–300 μm or 300–500 μm), which are then impregnated with the chemotherapeutic agents within onco-pharmacy, are being used to inject into the tumor. Other liver neoplasms treated by TACE include primary angiosarcoma or metastases from different primary tumors (Malogolowkin et al. 2000; Escobar Jr. et al. 2017; Cazejust et al. 2010; Mutabagani et al. 1999). A potential role of TACE is the treatment of PRETEXT I and II hepatoblastoma as neoadjuvant or adjuvant therapy to increase necrosis and potentially reduce relapse (alone or in combination with systemic therapy). Tumor necrosis, following neoadjuvant treatment, serves as a predictor for patient survival, especially in hepatoblatomas with undifferentiated small cells (Venkatramani et al. 2012). In the PRETEXT III or IV, TACE could be used to increase necrosis and hypothetically reduce relapse when used alone or in combination with systemic therapy.

### Trans arterial radio embolization (TARE)

This involves selective catheter implantation of yttrium-90 (^90^Y), which are β radiation (0.97mEv)–emitting radioisotopes, directly into the tumor mass by means of glass (Theraspheres; MDS Nordion, Ottawa, Canada) or resin (SIR-Spheres; Sirtex, North Sydney, Australia) microspheres. This provides targeted brachytherapy without the systemic side effects of radiation. This technique is frequently used to treat primary and secondary liver tumors in adults. Yttrium-90 decays to stable zirconium-90 with a physical half-life of 64.2 h. The mean tissue penetration of the energy emitted (average 0.9367 MeV, Maximum 2.27 MeV) is 2.5 mm with a maximum of 11 mm(Lungren et al. 2018). This application, although limited in availability, has been used in the pediatric setting (Hawkins et al. 2013). Alternatively, Holmium-166 can also be used to perform radioembolization, however there is no report of its use in children. This treatment modality could be considered in patients who do not have a chemotherapy-sensitive tumor or when toxicity limits have been reached from previous chemotherapy. It is critical to perform preliminary planning prior to radioembolization which involves appropriate patient workup and preparation, arterial mapping studies, embolization of nontarget collaterals and evaluation of pulmonary shunt fraction. In selected settings, there may be role for preoperative portal vein embolization to promote hypertrophy of the liver prior to partial hepatectomy by radioembolization. At the time of this publication, only 12 patients treated with radioembolization for liver tumors has been reported (Hawkins et al. 2013; Aguado et al. 2019). Potential indications of radioembolization in children may include patients with non-resectable liver malignancies such as hepatoblastoma, hepatocellular carcinoma or metastases as palliation or with curative intent or as a bridge to liver transplantation (Lungren et al. 2018).

Portal vein embolization has been used to promote the growth of the future liver remnant when insufficient liver volume can be predicted before hepatectomy in liver tumors. This has been used in toddlers with hepatoblastoma and mesenteric hamartoma to aid trisegmentectomy and hepatectomy by embolizing with the tumor feeding vessels with PVA and ethylene-vinyl alcohol copolymer (Onyx 18, ev3, France) respectively (Le et al. 2018; Terraz et al. 2015).

## Renal and suprarenal tumors

Wilms tumor is the most common malignant renal tumor of childhood accounting for accounts for about 6% of all malignant tumors in children. These tumors can be unresectable when tumor diameter is greater than or equal to 10 cm, when there is involvement of adjacent vital structures and if there is intracaval/atrial extension of the tumor. Pre-operative TACE with short term systemic chemotherapy with emulsion of pirarubicin, vindesine, cisplatin has been shown to be helpful in treatment of unresectable, metastatic, or diffuse anaplastic histology Wilms’ tumor with higher rates of complete tumor resection and relapse-free survival (Li et al. 2016; Li et al. 2011). Tumor necrosis was > 90% in 14 (25.5%), 50%–90% in 23 (41.8%), and < 50% in 18 cases (32.7%). 5-year event-free survival was 92.7%, and the overall survival was 94.5%. No drug-induced cardiotoxicity, nephrotoxicity or hepatic dysfunction was observed. Bland embolization has been performed in Wilm’s tumor to control hematuria after biopsy or treat life-threatening hemorrhage prior to nephrectomy (Smith et al. 2005; Chitnis et al. 2004).

Renal angiomyolipomas (AML) at a younger age, is aggressive and typically represents a component of tuberous-sclerosis-complex (Anis et al. 2020). The tumor comprising of fat and smooth muscle elements have a more than 50% risk of rupture and hemorrhage when greater than 4 cm in diameter (Rimon et al. 2006). Transcatheter renal artery embolization can be performed for hemorrhage from angiomyolipomas eliminating the need for blood transfusion and surgery in technically successful cases and, decrease tumor size in non-hemorrhagic AML (Hamlin et al. 1997; Dabbeche et al. 2006; Bardin et al. 2017; El Rafei et al. 2015). Transarterial embolization can be considered a safe primary option for symptomatic or larger AML (Anis et al. 2020). In patients with hematuria from renal tumors, such as, malignant rhaboid tumor, embolization can be performed to stop bleeding and facilitate chemotherapy (Sharma et al. 2013).

Similar to Wilm’s tumor, presurgical embolization can be performed in large neuroblastomas to reduce intraoperative bleeding at resection, treat bleeding after biopsy and shrink the tumor to decrease intraabdomial pressure and improve respiratory status (Krauel et al. 2009; Kyo Jin et al. 2018; Pio et al. 2017). A maximum primary tumor diameter > 13.20 cm and *MYCN* gene amplification were two independent risk factors for high-risk NB tumor rupture (Qin et al. 2020).

## Bone and soft tissue tumors

**Fig. 2:**
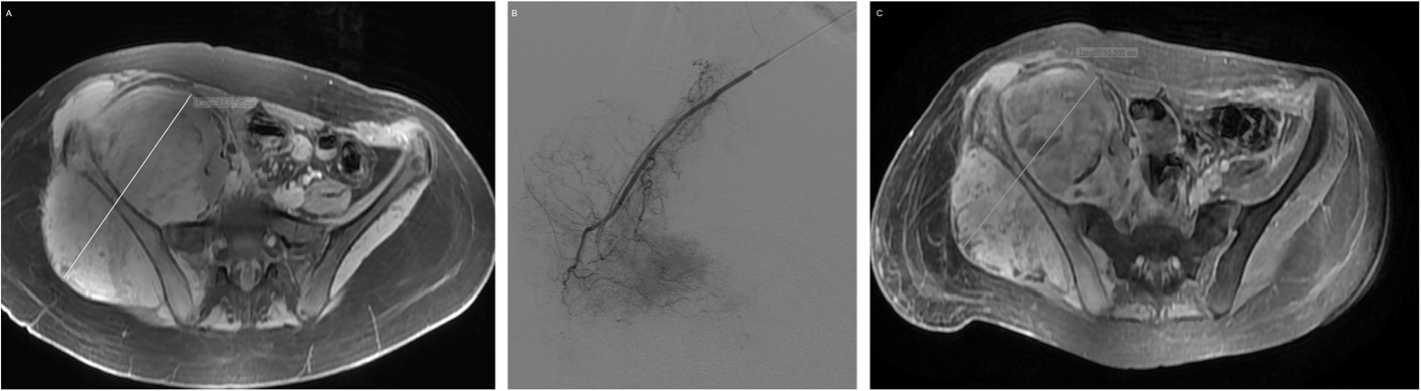
**a** T1-weighted fat saturated gadolinium (gd) axial MR image demonstrating a large pelvic osteosarcoma (biopsy proven) in a 14-year-old female. **b** Digital subtraction angiography demonstrating arterial tumor blush from branches of the internal iliac artery. These feeders were embolized with 100–300 DC beads loaded with 100 mg of doxorubicin (DEBDOX-TACE). **c** T1-weighted fat saturated gadolinium (gd) axial MR image at 1-month demonstrating reduction in tumor size with intra-tumoral necrosis

**Fig. 3:**
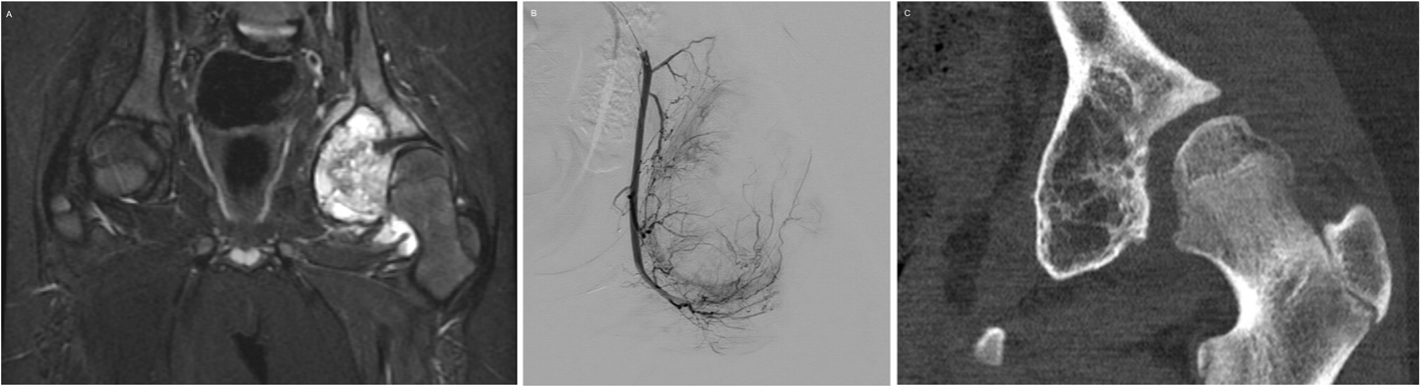
**a** Short tau inversion recovery (STIR) weighted coronal MR image demonstrating an expansile aneurysmal bone cyst of the left acetabulum in a 10-year-old male. **b** Digital subtraction angiography demonstrating arterial tumor blush from branches of the obturator artery. These feeders were embolized with 25% N-butyl cyanoacrylate and Lipiodol mixture. **c** Follow-up coronal CT image obtained 24-months after embolization demonstrating decrease in size of the ABC with ossification

Malignant bone tumors account for 6% of all childhood malignancies, 56% of which are osteosarcomas and 34% are Ewing sarcomas with peak incidence at 15 years of age (Gereige and Kumar 2010). Bland embolization can be used, both for local control and symptom palliation, in bone tumors (Mavrogenis et al. 2014). Surgery remains the standard treatment for osteosarcomas with adjunctive systemic chemotherapy, but transarterial chemoembolization in combination with limb salvage surgery yielded encouraging results when used to treat 32 patients with osteosarcoma prior to limb salvage surgery (Owen 2010) (Fig. 2). Chemoembolization with intra-arterial methotrexate has been used to treat resistant osteosarcoma (Avritscher and Javadi 2011). Preoperative embolization has proven to be an effective therapy in giant cell tumors, aneurysmal bone cysts, osteoblastomas, chondrosarcomas and vertebral hemangiomas to reduce blood loss and decrease transfusion requirements (Jha et al. 2016; Owen 2010; Pazionis et al. 2014; Ibrahim et al. 2013; Li et al. 2008; Boztug et al. 2006). Aneurysmal bone cysts (ABC) are benign bone tumors of childhood and early adulthood presenting as expansile osteolytic lesions with a varying potential to be locally aggressive. Embolization has been used for the treatment of ABCs, especially in locations where surgical management is difficult and associated with a substantial risk for complications, and also to minimize bleeding during excision (Rossi et al. 2010; Amendola et al. 2013; Donati et al. 2011; Boriani et al. 2014; Pearl et al. 2012) (Fig. 3).

**Fig. 4:**
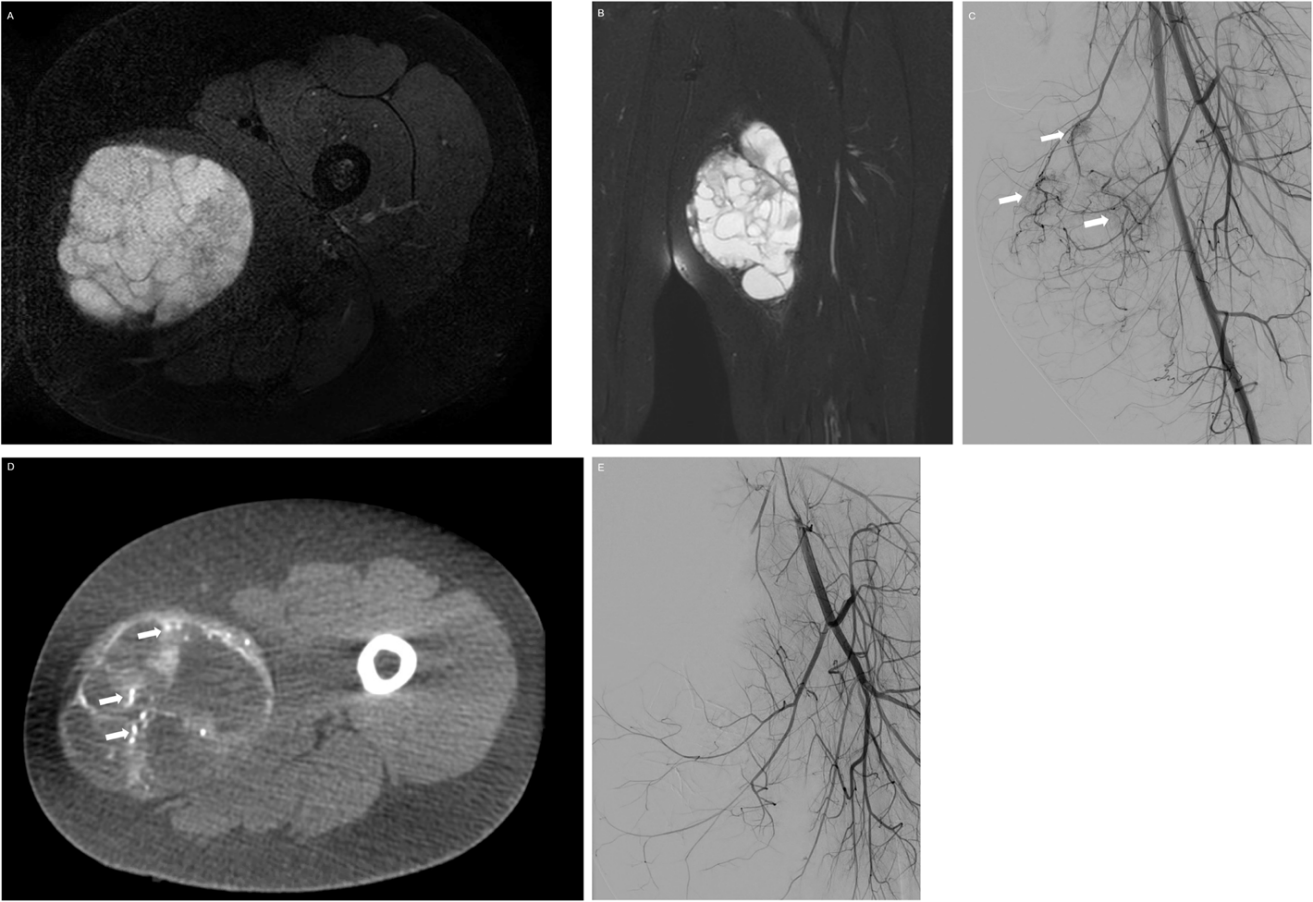
T2- weighted fat saturated axial (**a**) and coronal (**b**) MR image demonstrating a soft tissue sarcoma (malignant hemangiopericytoma) in the thigh of a 10-year-old male. A pre-resection embolization was requested due to its hypervascularity and risk of bleeding during surgery. **c** Pre-operative angiography demonstrating arterial tumor blush from branches of the superficial and deep femoral arteries (arrows). These feeders were embolized with 70–150 DC bead LUMI to reduce bleeding during resection. **d** Axial cone beam CT image obtained post embolization demonstrating the radio-opaque embolization beads in the tumor bed (arrows). **e** Post embolization angiography demonstrating disappearance of the arterial tumor blush

Doxorubicin administered by means of eluting beads for extra-abdominal desmoid tumors has shown promising results, among 4 children suffering recurrent or refractory desmoid tumors. This approach demonstrated reduction of tumor volumes ranging from 54% to 97% over a follow-up interval of 6–32 months (Elnekave et al. 2018). Also, neoadjuvant intra-arterial infusion of cisplatin, pirarubicin, and vindesine for rhabdomyosarcoma and endodermal sinus tumor has been performed (Wu et al. 2016). Bland embolization has been used in metastatic paraganglioma, myofibroblastic tumor, osteosarcoma and undifferentiated sarcoma to help in preparation for surgery, treat life-threatening event and for palliative care (Krauel et al. 2009) (Fig. 4). In very young infants, large (> 10 cm), sacrococcygeal teratomas pose risk of rupture and profuse bleeding before or during resection in whom preoperative embolization has been safely performed to mitigate the increased bleeding risk (Lahdes-Vasama et al. 2011; Cowles et al. 2006; Stavropoulou et al. 2019).

## Intracranial tumors

Hypervascular pediatric brain tumors such as choroid plexus papilloma, meningioma, astrocytoma, hemangioblastoma, yolk sac tumors and skull base tumors can be embolized pre-operatively to reduce blood loss (Wang et al. 2013; Amuluru et al. 2016). Currently a phase I/II trial is on for evaluating the role of intra-arterial infusion of Cetuximab and Bevacizumab for the treatment of relapsed or refractory intracranial gliomas in patients below 22 years (Health 2013).

## Retinoblastoma

Intra-arterial infusion chemotherapy with melphalan as the chemotherapeutic agent, is well-established for the treatment of retinoblastoma (Manjandavida et al. 2019). Targeted treatment in retinoblastoma by direct delivery of chemotherapeutic agents into the ophthalmic artery (OA) has dramatically changed the approach in the management of this deadly, yet curable eye cancer (Shields and Shields 2010; Anderson et al. 2017; Wyse et al. 2016). Inomato and Kaneko, in their initial series, used melphalan as single-agent chemotherapeutic drug for IAC (Inomata and Kaneko 1987). Abramson et al. added carboplatin and topotecan, which initiated the triple-drug regimen (Abramson et al. 2008; Gobin et al. 2011).

## Miscellaneous

Six children with different malignancies (acute lymphoblastic leukemia, acute myelogenous leukemia, chronic myelogenous leukemia, T-cell prolymphocytic leukemia, Langerhans cell histiocytosis, and rhabdomyosarcoma) had selective internal iliac arterial embolization with gelfoam and coils for hemorrhagic cystitis following stem cell transplantation (Bae et al. 2011).

## Future directions

Combined techniques, which use endovascular approaches for drug delivery with percutaneous tumor ablation techniques are being trialed in adults to test for greater treatment efficacy (Liapi and Geschwind 2007). If successful, such techniques could potentially be applied in pediatric patients. Immunoembolization is being trialed for metastatic uveal melanoma in adults, which similarly, could be expanded to pediatric patients (Institute 2018). Nano-particles coated with chemotherapeutic drugs or genetic agents could be delivered via endovascular route into the tumor. Certain specific aspects of the tumor, including immune response, oncogenic expression, susceptibility to chemotherapeutics, or its genetic potential, may be altered using these techniques.

## Conclusion

The lack of substantial evidence is one of the major drawbacks for wider acceptance of pediatric endovascular interventional oncological treatments. Creation of large multi institutional registries and repositories can help provide data base to study the efficacy and safety of these treatments on a larger scale. Additionally, involvement and partnership with organizations such as the Children’s Oncology Group, participation in clinical trials such as the pediatric hepatic international tumor trial (PHITT) in collaboration with other pediatric oncology care specialties would add value to this subspecialty. Creation and standardization of practice protocols and post treatment assessment indices will be necessary to substantiate the role of pediatric IO. However, pediatric care givers need to be aware of this modality so that it can be complemented with other cancer treatment modalities such as radiation, medical and surgical oncology in the wider management of pediatric cancers. This will help create a referral base to allow collection of additional experience in this field and create controlled prospective studies that will add to current knowledge on endovascular treatment in pediatric cancers.

## Data Availability

NA.
